# Does Implicit Self-Reference Effect Occur by the Instantaneous Own-Name?

**DOI:** 10.3389/fpsyg.2021.709601

**Published:** 2021-10-04

**Authors:** Ken Yaoi, Mariko Osaka, Naoyuki Osaka

**Affiliations:** ^1^Research Center for Child Mental Development, Kanazawa University, Kanazawa, Japan; ^2^United Graduate School of Child Development, Osaka University, Suita, Japan; ^3^Department of Psychology, Graduate School of Letters, Kyoto University, Kyoto, Japan; ^4^Center for Information and Neural Networks (CiNet), National Institute of Information and Communications Technology, Suita, Japan

**Keywords:** self-reference effect, recognition, implicit, own-name, self-representation

## Abstract

Self-reference effect (SRE) is defined as better recall or recognition performance when the materials that are memorized refer to the self. The SRE paradigm usually requires participants to explicitly refer items to themselves, but some researchers have found that the SRE also can occur for implicitly self-referenced items. Few studies though have investigated the effect of self-related stimuli without awareness. In this study, we presented self-related (participants’ names) or other (other’s names or nouns) stimuli for a very short time between masks and then explicitly presented subsequent trait adjectives to participants. Recognition performance showed no significant differences between the own-name and the other two conditions in Experiment 1 that had random-order conditions. On the other hand, the result of Experiment 2 that had block-order conditions and greater prime stimuli suggests that SRE can occur as a result of the instantaneous stimulus: Subjects who showed better memory performance also had relatively high recognition of the trait adjectives that they viewed after their instantaneously presented own-name. This effect would show that self-representation can be activated by self-related stimuli without awareness and that subsequent items are unconsciously referenced to that self-representation.

## Introduction

In our daily lives, we often prioritize the perception and processing of self-related stimuli over stimuli unrelated to the self. For example, we can automatically and quickly pick up (attend to) our own name in conversation even if we are at a raucous party – the so-called “cocktail party effect” (Wood and Cowan, 1995). Previous studies have found, in line with this effect, that we treat self-related stimuli in a special way (Bundesen et al., 1997; Shapiro et al., 1997; Devue and Bredart, 2008; Keyes and Dlugokencka, 2014). Additionally, prior cognitive functions bias our memory for self-referenced items, a phenomenon known as the self-reference effect (SRE; Greenwald and Banaji, 1989). The SRE is defined as better recall or recognition performance when the memorized materials refer to the self. For example, when participants judge words that are descriptive of themselves, they recall or recognize those words better than words that describe others. To date, various hypotheses about the SRE cognitive mechanisms have been proposed (Symons and Johnson, 1997). For example, Rogers et al. (1977) suggested that self-reference judgments produce a rich encoding unit that can function effectively during information processing. Furthermore, Bellezza (1984) argued that SRE occurs because the self provides a set of organized internal cues, and the materials associated with the cues are easier to retrieve during recognition/recall tasks. Symons and Johnson (1997) have reviewed many studies of SRE and have found that this effect results primarily because the self is a well-developed and often-used construct in memory that promotes the retrieval of encoded information. Furthermore, many recent neuroimaging studies using self-referencing tasks have found evidence suggesting that self-referential processing depends on a relatively unique neural basis specific to the ventral part of the medial prefrontal cortex (VMPFC), insula, and some other brain regions (Qin and Northoff, 2011; Denny et al., 2012; Wagner et al., 2012). These above-mentioned studies suggest that the SRE is a relatively special process itself; however, the details of the cognitive and neural mechanisms of SRE remain unclear.

Almost all of the investigations, however, of these psychological and neuroimaging studies have been made exclusively using explicit self-reference paradigm; that is, participants were required to judge how well they thought each word described themselves (or the other person), and then to consciously refer to the self- (or the other-) representation. However, like the cocktail party effect, we often process self-related stimuli automatically and unconsciously. Based on this premise, the cognitive function of our memory-based self-recognition would consist of both conscious (explicit) and unconscious (implicit) processes (Lieberman et al., 2004; Panksepp and Northoff, 2009), and it is possible that unconscious self-referenced processing also affects memory performance of the referenced items. Therefore, to reveal the cognitive or neural basis of SRE and self-recognition processes in more detail, we consider that it is necessary to employ both an explicit and an implicit self-reference paradigm. Prior to this study, some researchers found that the SRE also occurs for implicit self-referenced items (Cloutier and Neil Macrae, 2008; Cunningham et al., 2008; Turk et al., 2008; Kesebir and Oishi, 2010; van den Bos et al., 2010). For example, van den Bos et al. (2010) used an “ownership” procedure to investigate whether less evaluative forms of self-referential cognition produce some advantages in the memory performance. In their experiment, participants were required to sort items into virtual baskets that belonged to themselves or to a fictitious other, and they showed better recognition performance for the items encoded in the context of self-ownership than for those items encoded in other-ownership. They suggested that creating a context of self-referential encoding leads to elaborative representations in memory, even in the absence of explicit self-evaluation. Furthermore, Kesebir and Oishi (2010) investigated participants’ memory performance regarding others’ birthdays and found that they were more likely to remember another person’s birthday if the date was close to their own birthday. This was true even when participants were not required to refer to their own birthday and when the self-related information (self-cues) was not presented explicitly. From this result, they also suggested that SRE can occur spontaneously in the absence of explicit self-referenced cues if the items themselves that are to be learned automatically activate self-relevant information. These studies showed that people associate items with self-representation and remember them well without explicit referential processing or self-cues.

In addition to those studies pertaining to the SRE, a few studies have investigated the effect of perceiving self-related stimulus without awareness. Using the subliminal self-face, Geng et al. (2012) found that electrophysiological activities were directly evoked by the subliminal self-face, suggesting rapid, low-resource-consuming self-face processing. Similarly, the study by Tao et al. (2012) indicated that participants showed a self-positivity bias by the subliminal self-face with an interocular suppression technique. These results suggest that self-related information evokes implicit self-evaluative processes, even when presented subliminally, and that those processes work as a special prime stimulus for another subsequent stimulus (Tao et al., 2012). Furthermore, in recent years, several studies have supported the perception or processing of the self-relevant stimuli without awareness in terms of neural activity (Rameson et al., 2010; Zhou et al., 2017; Xia et al., 2021). For example, Xia et al. (2021) measured EEG during the task in which participants rated positive or negative adjectives after subliminally presenting their own-names. The results showed that the response to positive adjectives was faster than that to negative adjectives in the own-name condition. In addition, both the latency and the amplitude of N400 showed a significant interaction between the name-cue and emotional valence.

Based on this standpoint, we hypothesized that the items presented after instantaneous self-related information would be memorized better than the other-related information, that is, SRE would occur. In this regard, exploring both explicit and implicit self-evaluative processes that cause SRE is necessary to reveal how memory-based self-recognition works based on both conscious and unconscious processes.

In the present study, therefore, we conducted two experiments with different parameters and designs to investigate whether the SRE occurs by implicit self-referential processing evoked by the self-related stimulus that was presented without participants realizing. We focused on participants’ own name as the self-related stimulus because many studies have been done that have indicated special features of the own-name just like own-face (Shapiro et al., 1997; Tacikowski et al., 2011, 2013; Zhao et al., 2011; Yamada et al., 2012; Nakane et al., 2016). For example, Shapiro et al. (1997) indicated that participants could detect their own name better as a second target in a rapid serial visual presentation (RSVP) stream than another person’s name or a noun. These studies suggested that own-name is more likely to capture attention (and be detected automatically) than other names and to activate self-representation.

In our experiments, participants were explicitly required to perform an evaluation task that was irrelevant to themselves, but where each evaluated item was actually presented after an instantaneously presented participants’ own name, another person’s name, or a noun between mask stimuli. If the instantaneously presented own-name activated self-representation without awareness and implicitly drove self-evaluative processes, then the subsequent items were memorized better relative to other two conditions.

## Experiment 1

### Materials and Methods

#### Subjects

Thirty healthy Japanese participants (18 males; mean age=22.2±4.2years.) were recruited in this research study.^1^ All participants had normal or corrected-to-normal vision. Both Experiments 1 and 2 were conducted in accordance with the ethical guidelines of the Center for Information and Neural Networks (CiNet, Osaka, Japan). All participants provided written informed consent prior to the experiment. Participants were rewarded with a 1,000 Japanese yen library card at the end of the experiment. Three participants were excluded from the analysis; one participant reported that both his first and last names belonged to his acquaintances in the other-condition, and other two participants might not be able to perform the task correctly (their false alarm rate >70%).

#### Stimulus

For the evaluation and recognition tasks, 300 Japanese trait adjectives were selected from the corpus of Aoki (1971), who examined the desirability ratings of 455 words. All trait adjectives were written with two to nine characters in Japanese. From these words, we selected the top 96 high-desirability (2.0–4.2), the bottom 96 low-desirability (6.2–7.9), and 96 mid-desirability (4.4–6.2) words. We then divided each 96-word list into 12 groups (eight high-, eight mid-, or eight low-desirability words) as their average desirability and word length were nearly equal. We then combined high-, mid-, and low-desirability groups and made 12 word lists (each including eight high-, eight mid-, and eight low-desirability words). Six of the 12-word lists were used for the evaluation task, and the other six were used as fillers for the recognition task. We counterbalanced the allocation of the word lists across participants and conditions. Furthermore, another six words were selected for the practice of the evaluation task.

In the evaluation task, we used the participant’s own name, another participant’s name, or a noun as prime stimuli. Each word had from two to five characters (with a width of about 3.0°–7.3° and a height of about 1.4° of visual angle) and was written in Japanese *kanji*, which uses ideogrammatic characters. The Japanese participants likely had strong familiarity with each *kanji* character in their own name because each character is associated with a particular meaning and because they were likely to have each read and written their own names in *kanji* since their childhood. Therefore, we hypothesized that participants would recognize their own name without awareness even when the order of characters in their name was permutated, and also that if the instantaneously presented own-name triggered the SRE, there would be equivalent effects even when presenting the name in a different order. For this reason, we also used the name and noun with the reordered characters as prime stimuli.

Furthermore, to mask these prime stimuli, black-and-white checkerboard patterns were presented as mask stimuli. The sizes of these stimuli were 10.0° wide and 1.9° high at visual angles, which could cover the entire area of the prime stimuli.

#### Procedure

In order to perform the evaluation and recognition tasks, each participant sat in front of a 24-inch LCD screen (refresh rate: 60Hz) and fixed his or her head position 50cm away from the display using a chinrest.

**Figure 1:**
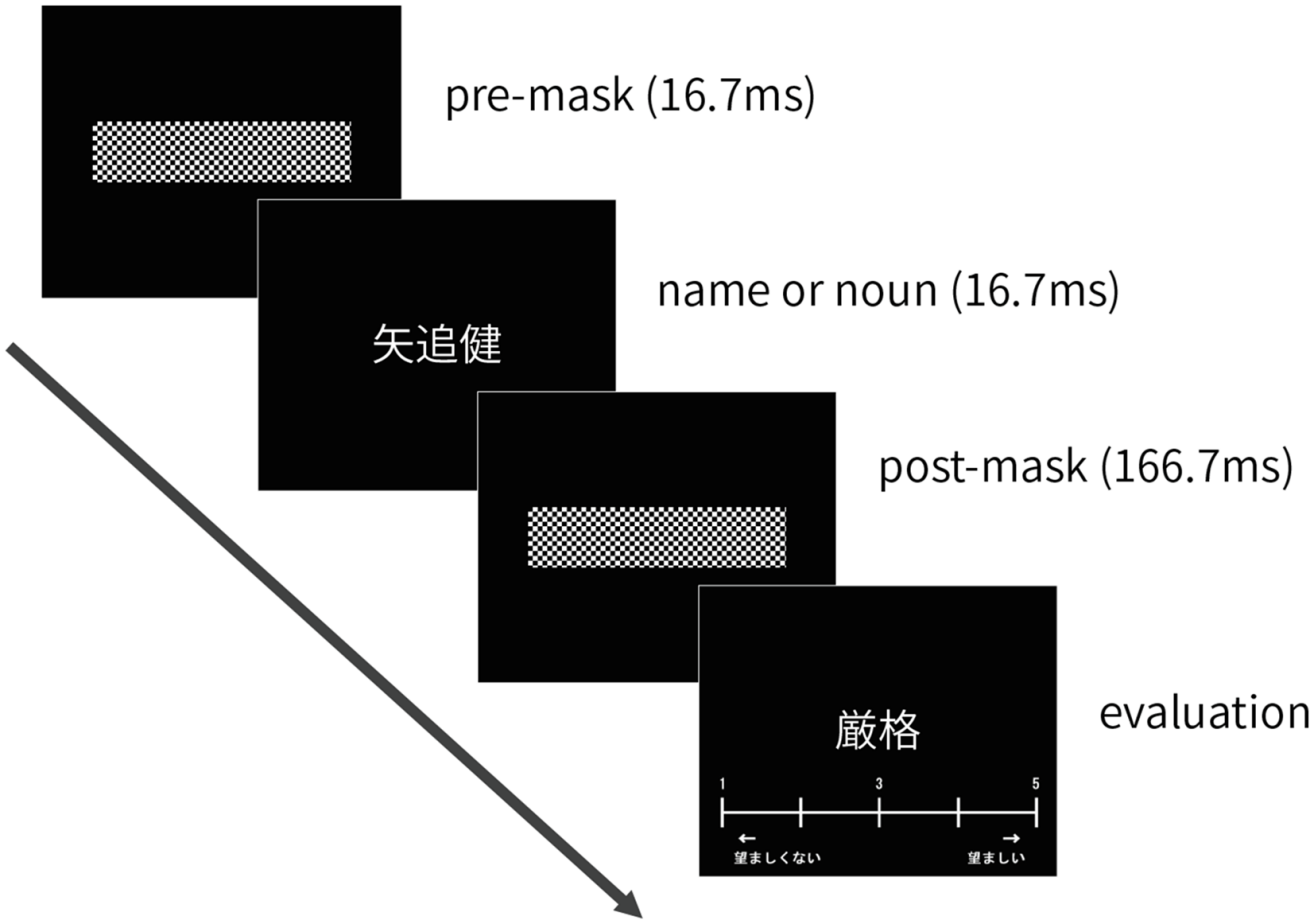
The sequence sample of one trial on the evaluation task in Experiment 1. Participants were required to fix their eyes on the center of the screen and to evaluate the social desirability of each trait adjective.

In the evaluation task, participants were required to fix their eyes on the center of the screen and to evaluate the social desirability of each trait adjective. They were unaware that each adjective was presented after a prime stimulus. In each trial, a name or a noun (16.7ms) was flashed in the center of the screen between a pre-mask (16.7ms) and a post-mask stimulus (166.7ms). Just after the post-mask stimulus, a trait adjective and rating scale were presented, and participants judged the social desirability of each word on a five-point scale without a time limit and using the keyboard (Figure 1).

In the “self” and “self-shuffle” conditions, participants’ own names or permutated names, respectively, were presented as the prime stimulus. Similarly, in the “other” and “other-shuffle” conditions, the name of another participant in this experiment was presented in its normal or permutated form. Each of the names of participants was used once in the “other” condition in this experiment. Furthermore, in the “word” and “word-shuffle” conditions, a neutral noun (e.g., “document”) in *kanji* or a permutated noun was presented. All these stimuli had the same number of characters (from two to five, depending on the number of each participant’s own-name) and did not contain the same character (*kanji*) as the others. The order of these conditions was randomized for each participant. Prior to the evaluation task, participants performed a practice task. Using a procedure similar to that of the real task, six conditions contained one word each. All of the evaluation tasks and recognition tasks in this study were performed using Presentation software (Version 18.3, Neurobehavioral Systems, Inc., Berkeley, CA, United States).

Between the evaluation task and subsequent recognition task, participants performed a mental arithmetic task to set retention delay and to prevent participants from verbally rehearsing words: they were required to subtract seven words from 1,000 successively for 5min.

After the mental arithmetic task, participants performed the recognition task. They were required to respond “New,” “Know,” or “Remember” to 288 words without a time limit and using the keyboard, with 144 words having been used in the evaluation task and 144 serving as distractors. In this study, we employed the Remember-Know paradigm because previous studies have indicated that memory performance of a “Remember” judgment is a solid index for reflecting self-referential cognition (Conway and Dewhurst, 1995; Conway and Pleydell-Pearce, 2000). Participants were required to answer “New” when they did not see the word in the evaluation task. Otherwise, they answered “Remember” when they could consciously recall having seen the word and could retrieve any information about this event, or “Know” when they recognized the word purely on the basis of a feeling of knowing that the word had been presented and could not recall any further details. Because we aimed to investigate the incidental learning of words used in the evaluation task, participants were not informed about this task prior to the experiment.

Finally, participants were asked whether they could detect any prime stimulus (someone’s name or noun) before each trait adjective, and whether each first and last name used in the “other” condition accorded with the name of their friend or acquaintance.

### Results

In the evaluation task, no participants reported that they could detect any instantaneously presented stimulus (name or noun) before the presentation of each trait adjective. To compare the recognition performance between conditions, we calculated *d’* from the hit and false alarm rate in the recognition performance (Table 1). Results show that the performance of the self (and also word-shuffle) condition was slightly higher than the performance of the others. However, three (conditions: self vs. other vs. word)×two (spelling: normal vs. shuffle) ANOVA showed no significant main effect [*F* (2, 52)=0.88, *ƞ*^2^=0.004; *F* (1, 26)=0.09, *ƞ*^2^<0.001; *ps*>0.05] or interaction [*F* (2, 52)=1.59, *ƞ*^2^=0.007; *p*>0.05] across factors. In addition, to investigate the effect of the desirability of trait adjectives on recognition performance, ANOVA was conducted with the desirability (positive, neutral, negative) as a factor, and a significant main effect of the desirability was found [*F* (2, 52)=20.55, *ƞ*^2^=0.10]. The *post hoc* multiple comparisons of Shaffer’s Modified Sequentially Rejective Bonferroni Procedure indicated that the recognition performance of the positive adjectives was significantly lower than that of other two (neutral, negative) conditions (*ps*<0.01). This result may be due to the higher false alarm for positive adjectives. On the other hand, there was no significant interaction between desirability and other factors (*ps*>0.05). Furthermore, any other analysis (e.g., the percentage of “Remember” responses in hit trials; Table 2) also showed no significant effects and interactions (*ps*>0.05). Therefore, the effect of an own-name that was presented without participants realizing was not confirmed in Experiment 1.

**Table 1:** Mean recognition performance (*d’*) and SD for each condition on the recognition task in Experiment 1 (*n*=27).

	Self	Self-shuffle	Other	Other-shuffle	Word	Word-shuffle
*d’*	2.00	1.97	1.93	1.88	1.88	2.00
*SD*	0.48	0.52	0.48	0.46	0.51	0.45

**Table 2:** Mean percentage (%) and SD of remember response on hit trials (number of remember responses/number of hit trials) of the recognition task in Experiment 1.

	Self	Self-shuffle	Other	Other-shuffle	Word	Word-shuffle
Remember (%)	55.3	56.5	57.0	61.3	58.0	55.7
*SD*	24.9	26.3	23.8	24.2	27.8	26.2

### Discussion

In this experiment, we hypothesized that the words presented after an instantaneous own-name between masks would be better memorized than would the words after the presentation of another participant’s name or a noun. In the evaluation task, all participants reported that they could not detect any self-related stimulus before each trait adjective. We are not able to conclude that the instantaneously presented name and noun in this experiment were as truly “subliminal” stimuli for participants because we had relied on participants’ single subjective reports at the end of the task. At least, however, we consider it is unlikely that participants consciously referred to the self or the other when they judge the social desirability of each word. On the other hand, while participants did show slightly higher memory performance in the self-condition, there were no significant differences across conditions.

Conway and Dewhurst (1995) indicated in their study that the memory performance of a “Remember” judgment should be a solid index for reflecting self-referential cognition, but that there were no significant differences for the percentage of “Remember” hit responses.

From these results, even if presenting an instantaneous own-name before each trait adjective has some sort of effect (thereby improving memory performance for subsequent trait adjectives), that effect is likely too weak to adequately influence memory performance. Whereas each condition was presented in random order in Experiment 1, the effect would possibly become greater if each condition was coordinated in a group (block design). Furthermore, it is possible that the own-name and other prime stimuli were presented too weakly and briefly to discern the impacts of stimuli, thus making the amount of self-related information insufficient to create the SRE. Therefore, we conducted Experiment 2 by changing the experimental design and some parameters in the evaluation task of Experiment 1.

## Experiment 2

### Materials and Methods

#### Subjects

Thirty-three healthy Japanese participants (17 males; mean age=20.9±2.1years.) were recruited (none of the participants had participated in Experiment 1). All participants had normal or corrected-to-normal vision. Participants were rewarded with a 1,000 Japanese yen library card at the end of the experiment. One participant reported that both her first and last names belonged to her acquaintance, who was in the other-condition, and two participants did not use the experimental device correctly. Therefore, these participants were excluded from the analysis.

#### Stimulus

All trait adjectives and word lists were the same as in Experiment 1, but the sizes of prime stimuli were larger than in Experiment 1. They had from three to five characters (with a width of about 11.9°–20.2° and a height of about 3.7° of visual angle). Black-and-white checkerboard patterns (19.9° wide and 4.7° high) were presented to mask these stimuli.

#### Procedure

In the evaluation task, the duration of the prime and mask stimuli were lengthened: Each prime stimulus (33.3ms) was flashed longer than in Experiment 1 between a pre-mask stimulus (1,000ms) and a post-mask stimulus (33.3ms). Moreover, “self,” “other,” “word,” and corresponding shuffle conditions were arrayed in accordance with ABCCBA block design. Each condition had two blocks, and each block had 12 trials. We counterbalanced the sequence of conditions and the allocation of the word lists across all participants. Between the evaluation and the recognition tasks, participants performed a mental arithmetic task that consisted of many additions. They were required to add two numbers, each five digits in length, to each other as many times as possible in 10min. The methods for defining other parameters and procedures were the same as Experiment 1.

### Results

As in Experiment 1, no participants could detect any instantaneously presented stimulus (name or noun) before the trait adjectives. Table 3 shows the recognition performance (*d’*) under each condition in the recognition task. For these performances, three (self vs. other vs. word)×two (normal vs. shuffle) ANOVA showed no significant main effect [*F* (2, 58)=1.15, *ƞ*^2^=0.004; *F* (1, 29)=0.17, *ƞ*^2^<0.001; *ps*>0.05] or interaction [*F* (2, 58)=0.18, *ƞ*^2^<0.001; *p*>0.05] across conditions. In addition, ANOVA was conducted with the desirability (positive, neutral, and negative) as a factor, and a significant main effect of the desirability was found [*F* (2, 52)=13.4, *ƞ*^2^=0.06]. The *post hoc* multiple comparisons of Shaffer’s Modified Sequentially Rejective Bonferroni Procedure indicated that the recognition performance of the positive adjectives was significantly lower than that of other two (neutral and negative) conditions (*ps*<0.01), as in Experiment 1. On the other hand, there was no significant interaction between desirability and other factors (*ps*>0.05).

**Table 3:** Mean recognition performance (*d’*) and SD for each condition on the recognition task in Experiment 2 (*n*=30).

	Self	Self-shuffle	Other	Other-shuffle	Word	Word-shuffle
*d’*	2.03	2.01	1.92	1.97	1.93	1.95
*SD*	0.61	0.63	0.63	0.52	0.48	0.48

**Figure 2:**
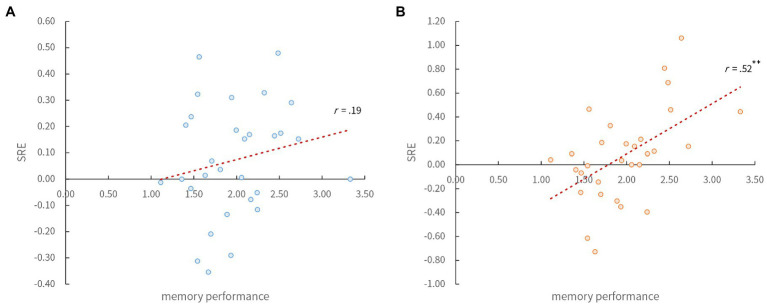
Scatter plot and Pearson’s correlation between memory performance and the self-reference effect (SRE) in Experiment 2. (**A**) Shows the index of the SRE to other-condition (difference between the *d’* in the self and other-condition; *y*-axis) and memory performance (mean *d’* of all conditions; *x*-axis). (**B**) Similarly shows the index of the SRE to word-condition (difference between the *d’* in the self and word-condition) and memory performance. The red dashed line indicates the linear regression line. ^**^p<0.01.

**Figure 3:**
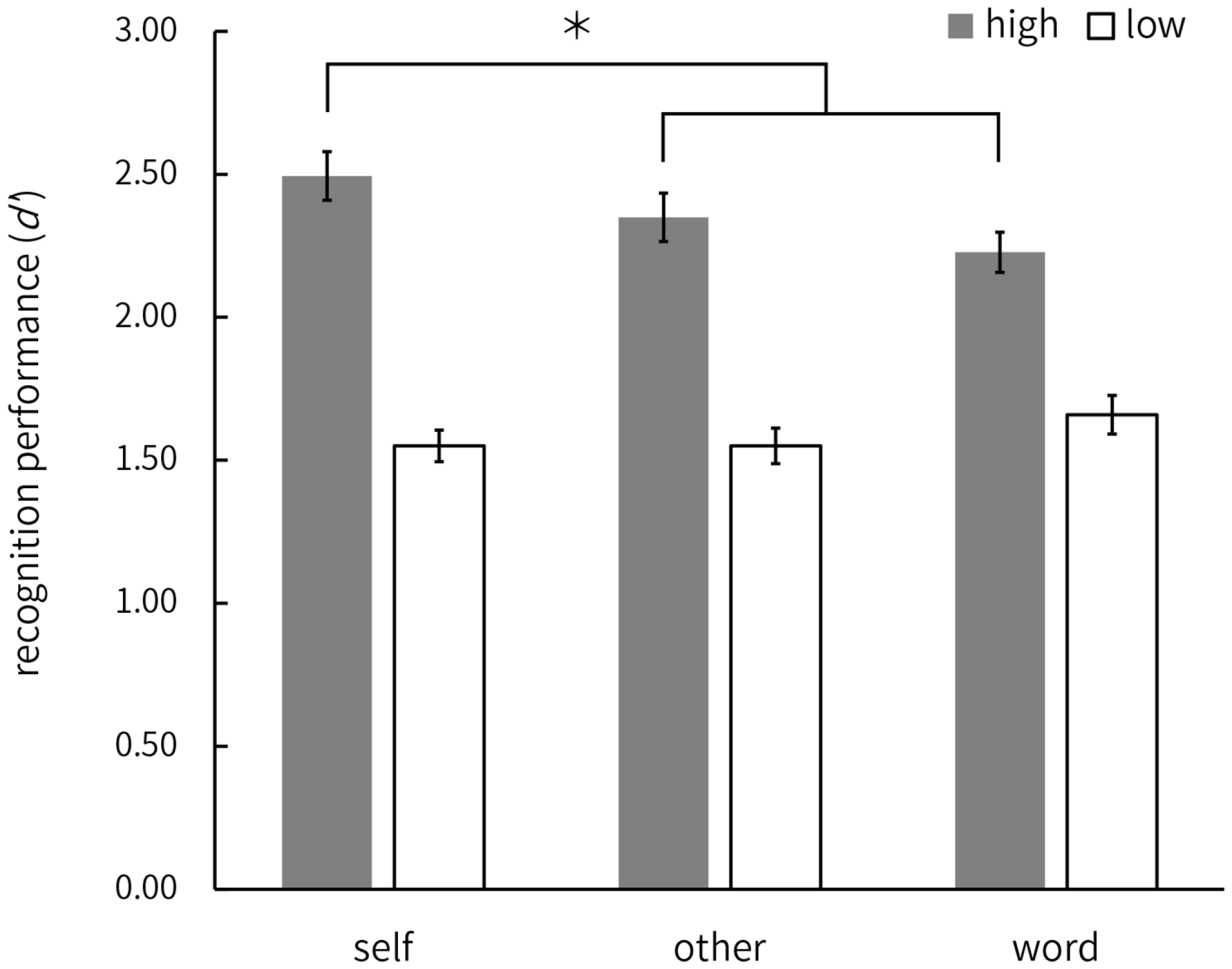
Mean recognition performance (*d’*) of high- and low-performance groups for each condition on the recognition task in Experiment 2. The factor of spelling (normal or shuffle) was combined. Error bars indicate standard error, ^*^p<0.05.

We then focused on individual differences in recognition performance because it was possible that the high performers had higher recall of the memorized words than did the low-performers, and perhaps they would have responded “Remember” more. Therefore, we investigated the possibility that the recall of the high performers was more affected, being driven by the self-related information even if they did not consciously refer to the self (Conway and Dewhurst, 1995; Conway and Pleydell-Pearce, 2000). For this purpose, we firstly investigated whether the participants with higher memory performance remembered the words associated with the self better. We calculated the mean *d’* of all conditions for each participant as an index of memory performance, and the difference between the *d’* in the self and each of the other two conditions as an index of the SRE. Pearson’s product–moment correlation coefficient between these two indices showed that there was no significant correlation between the SRE for the other-condition and memory performance (*r*=0.19, *p*>0.05; Figure 2A), but there was a significant correlation between the SRE for the word-condition and memory performance (*r*=0.52, *p*<0.01; Figure 2B). This result suggests that the SRE for words associated with instantaneously presented own-name is greater in participants with higher memory performance. Furthermore, when we divided participants into two groups according to the mean *d’* of all conditions, there were differences in the number of “Remember” responses between the upper (high-performance group; *n*=15) and the lower half of participants (low-performance group; *n*=15; Table 4). In their memory performance, two (group: high vs. low)×three (conditions: self vs. other vs. word)×2 (spelling: normal vs. shuffle) ANOVA showed no significant main effect of conditions and spelling [*F* (2, 56)=1.37, *ƞ*^2^=0.004; *F* (1, 28)=0.17, *ƞ*^2^<0.001; *ps*>0.05], but it did show a significant main effect of groups [*F* (1, 28)=51.36, *ƞ*^2^=0.486; *p*<0.01] and interaction between groups and conditions [*F* (2, 56)=6.47, *ƞ*^2^=0.020; *p*<0.01]. The *post hoc* multiple comparisons of Shaffer’s Modified Sequentially Rejective Bonferroni Procedure indicated that the recognition performance of the self condition (combined spelling factor) was significantly better than that of other two (other, word) conditions in the high-performance group only (*ps*<0.05; Figure 3). These results indicate that the high-performance group (both normal and shuffle) memorized words after an instantaneously presented own-name better than other groups – that is, SRE occurred for the high-performance group.

**Table 4:** Mean number of remember and know responses on hit trials of high- and low-performance groups on the recognition task in Experiment 2.

		Self	Self-shuffle	Other	Other-shuffle	Word	Word-shuffle
Remember	High	13.20	12.67	11.53	11.73	11.47	10.80
	Low	8.60	8.60	8.27	9.40	8.60	9.07
Know	High	8.13	8.80	9.33	8.93	8.67	9.47
	Low	10.87	10.27	10.40	10.47	11.33	10.87

### Discussion

Based on the recognition performance of all participants, we also could not confirm improved memory of a word presented after an instantaneously presented own-name, such as in Experiment 1. The SRE is known to be relatively robust, but we assume that this effect is fairly small if the self-related information is not explicitly presented.

In Experiment 2, however, the high-performance group did show better recognition performance in the self (both normal and shuffle) condition than in the other and noun conditions. Previous studies (Conway and Dewhurst, 1995; Horiuchi and Fujita, 2001; Fujita and Horiuchi, 2004; van den Bos et al., 2010) have found that SRE does not occur with a “Know” response (reflecting familiarity without recollective experience) but does occur with “Remember” responses that reflect recollective retrieving experience for encoded items. It seems that the high performers were relatively more inclined to use recollective retrieval of words than were low-performers (and thus responded “Remember” more); therefore, they showed some advantages of memory performance in the self condition. This result indicates that the SRE can occur as a result of self-related information that activates self-representation without awareness and implicitly drives self-evaluative processes. As a result, participants in the self condition memorized the subsequent items better than did those in other conditions. Therefore, only the high performers who had the recollective experience of the items were likely to show the SRE (Conway and Dewhurst, 1995).

On the other hand, it has been suggested that the perception of the self-relevant stimuli is affected by working memory capacity (Conway et al., 2001; Naveh-Benjamin et al., 2014; Röer and Cowan, 2021). In general, it is known that participants with higher working memory capacity are less likely to notice the self-relevant stimuli (e.g., own-name) presented irrelevantly to the ongoing task (Conway et al., 2001). It means that it is more difficult for participants with lower working memory capacity to suppress attention-capturing stimuli such as own-name. However, the result of Experiment 2 seems to indicate that, on the contrary, participants with good memory performance were more affected by their own-name. There are several possible reasons for this result. One reason is that the higher memory performance in this study may not necessarily reflect the higher working memory capacity. It may be that the task in this study required only implicit memory and not working memory, which is important for attention control and task switching. The other reason is that the own-name in this experiment was presented so instantaneously that the participants could not detect it, and it was presented at different timing (inter-trials) from the word to be memorized. That is, participants with higher working memory capacity may have been more affected by their own-name because they were paying more attention to the screen between trials. In any case, further studies measuring the working memory capacity of each participant are needed to clarify this point.

## General Discussion

In this study, we investigated whether words presented after an instantaneous own-name between masks were memorized better than they were after another person’s name or after an unrelated noun – that is, whether SRE occurred. Recognition performance showed no significant differences between the own-name and the other two conditions in Experiment 1, but participants who had a relatively good performance in the recognition task showed in Experiment 2 some of the self-advantage effect on memory caused by the instantaneous presentation of their full name and their permutated name.

The two main procedural differences between Experiments 1 and 2 are as follows: (i) the order of conditions, random, or block order; and (ii) the amount of prime stimuli (size and duration). It appears that the one-time effect of the instantaneously presented own-name had a fairly small effect (if any). Therefore, it was difficult to dissociate how the different conditions in Experiments 1 and 2 affected memory performance differently. We expect that implicit self-evaluative processes are likely to be more present when the self-related information is repeated to a greater degree (i.e., bigger and longer) several times in a block, as in Experiment 2. In any case, improving the order or increasing the amount of self-related stimulus is likely a necessity to adequately result in the SRE.

In Experiment 2, only high performers showed the self-advantage effect on memory by instantaneously presented their own-name and permutated own-name. The study by Kesebir and Oishi (2010) and other studies have found that SRE can occur spontaneously, even in the absence of explicit self-cues or self-evaluation (Cloutier and Neil Macrae, 2008; Cunningham et al., 2008; Turk et al., 2008; van den Bos et al., 2010). These studies suggest that various self-relevant cues could drive increased elaborative encoding and make the performance of memory of peripheral items better than the memory of others. In this study, we did not find an overall general effect across all participants. However, we think that the better performance of the self condition in Experiment 2 is derived from cognitive processes that are related to implicit SRE. This study provides the first evidence for the memory-advantage effect of instantaneously presented self-related information for subsequent items.

In most of the previous studies that examined the SRE, participants were required to explicitly make judgments about themselves. However, some studies have suggested that the SRE can occur without explicit association with participants themselves (Cloutier and Neil Macrae, 2008; Cunningham et al., 2008; Turk et al., 2008; Kesebir and Oishi, 2010; van den Bos et al., 2010). A recent study, using a similar experimental paradigm as this study, suggested that the subliminal presentation of own-name can affect the subsequent response to adjectives in terms of reaction time and neural activity (Xia et al., 2021). The results of this study confirm these findings and also indicate that the own-name presented instantaneously may have the effect of causing the participants to better remember the words that appear after the own-name.

We have, however, several issues to resolve in order to unveil further details about this implicit SRE. First, it is possible that the effect of the own-name in the high-performance group was a result of using *kanji* characters because the permutated own-name had an almost equivalent effect on memory performance as did the nonpermutated own-name. *Kanji* is an ideographic character, and each character has its own meaning. For this reason, the amount of information per character is greater than that of phonetic characters, and the meaning of each *kanji* character does not change regardless of its order. Many Japanese people have read and written their own name in *kanji* repeatedly and understand the meaning of the characters in their own name. We consider that each *kanji* character in the own-name is more likely to become a self-related stimulus, and that participants recognized those characters as their own name even when the order of characters was permutated. Therefore, it is necessary to investigate the implicit effect of other types of self-related stimuli, such as self-face, which is known to evoke implicit self-evaluative processes even when presented subliminally (Geng et al., 2012; Tao et al., 2012). Secondly, we believe that individual or cultural differences may have influenced the effect in this study. For example, Chen et al. (2015) found that self-esteem could modulate automatic attention bias toward one’s own name. In line with that finding, participants’ self-esteem, attachment to their own name, or other cultural factors (e.g., the number of people who have same first and last names) may influence implicit self-evaluative processes driven by the own-name. Third, when examining the effects of self-relevant information like this study, it is important to note the effects of repeated presentation of the stimulus. Specifically, some previous studies suggested that repetitive presentation of own-name (visually or auditory) attenuated its effect, especially on attention (Harris and Pashler, 2004; Röer et al., 2013). However, at least in the present study, we consider that the effect of repeated stimuli may not have occurred or have been weak. This is because the participants did not notice that they were presented with stimuli related to themselves, and the SRE was not observed in Experiment 1, in which each condition was presented randomly, but was observed in Experiment 2, in which the conditions were presented in block order (repeated presentation). On the other hand, the effect of presenting more repetition of the self-related stimuli is not clear, therefore further study is needed.

In any case, we could not conclude whether the self-advantage effect in our experiments was based on the same cognitive processes as the typical SRE. Further study is needed to investigate details of the cognitive and neural basis of this effect. For example, a neuroimaging study would be able to show the neural correlates of perceiving instantaneous own-name unconsciously and subsequent items. Clarifying explicit and implicit self-evaluative processes would help to clarify similarities and differences between conscious and unconscious mechanisms of human self-recognition.

## Conclusion

In the current study, we investigated whether the SRE occurs by instantaneously presented self-related stimuli (participants’ own name) without awareness. Results show that the SRE did occur as a result of that self-related stimulus – that is, the high-performance participants had relatively high recognition of trait adjectives that were viewed after instantaneously presented own-name. This effect shows that self-representation is able to be activated unconsciously by some kind of self-related stimulus and that subsequently viewed items are implicitly referenced to that self-representation.

## Data Availability Statement

The raw data supporting the conclusions of this article will be made available by the authors, without undue reservation.

## Ethics Statement

The studies involving human participants were reviewed and approved by Center for Information and Neural Networks (CiNet). The patients/participants provided their written informed consent to participate in this study.

## Author Contributions

KY designed the experiment, analyzed the data, and wrote the manuscript. MO and NO supervised the study and gave advice regarding the experimental outline. All authors contributed to the article and approved the submitted version.

## Funding

This work was supported by the Japan Society for the Promotion of Science KAKENHI Grants #26730075 and #19K16893 to KY, #15H01690 to NO, and #18H03666 to MO.

## Conflict of Interest

The authors declare that the research was conducted in the absence of any commercial or financial relationships that could be construed as a potential conflict of interest.

## Publisher’s Note

All claims expressed in this article are solely those of the authors and do not necessarily represent those of their affiliated organizations, or those of the publisher, the editors and the reviewers. Any product that may be evaluated in this article, or claim that may be made by its manufacturer, is not guaranteed or endorsed by the publisher.

## Footnotes

^1^The sample size for the main effects of conditions in Experiment 1 and 2 were estimated using G*Power (version 3.1.9.4; Faul et al., 2009). We assumed a medium effect size (*f* = 0.25) and medium correlations (*r* = 0.5) among repeated measures. Furthermore, alpha and power (1-beta) were set to be 0.05 and 0.8, respectively (Cohen, 1992). This resulted in a required minimum sample size of 28.
